# The Halifax Union Poor-Law Hospital

**Published:** 1914-12-12

**Authors:** Henry Burdett


### REPORTS ON Hospitals of the United Kingdom.

### THE HALIFAX UNION POOR-LAW HOSPITAL.

By SIR HENRY BURDETT, K.C.B., K.C.V.O.

It is both interesting and instructive when an inspector who moves up and down the country comes in contact with those responsible for the administration of many hospitals. For he is able to note the individual force which continuously gathers weight from the formulation of a relatively perfect model and the example of a conscientious well-doing resting on sound principles in the administration of a particular hospital. At Halifax, as we reported in The Hospital on October 31, page 113, there is one of the most interesting and, on the whole, having regard to its date, one of the most excellent types of a well-constructed, carefully thought out, and efficient group of hospital buildings.

### The Voluntary Hospital's Example.

Our visit to the Halifax Union Poor-Law Hospital made it apparent that the example of the voluntary hospital had materially influenced the Guardians, who are responsible for the expenditure of so large a sum on so relatively perfect and well-planned a set of hospital buildings as that which constitutes this Union Poor-Law Hospital. The plan might have been selected in competition for a voluntary hospital of the first rank with 400 beds. The buildings are sound and good. The arrangements of the various units, with the exception of the operation unit, are well up to date, and their equipment is remarkable from the fact that so far as the sanitary blocks are concerned they contain some most expensive types of appliances, and include the newest and, on the whole, probably the best mackintosh bath available for hospital use. There are certainly few, if there are any, Poor-Law infirmary buildings which are better, if they are even as good from the technical aspect of hospital planning, than those of this Poor-Law hospital. The planning includes two blocks of circular wards, which contain accommodation for patients suffering from tuberculosis, and the patients in these wards are probably placed, either hygienically or otherwise, in more favourable conditions than those to be met with in the majority of sanatoria. Certainly they are, on the whole, in our opinion, the most attractive and the best type of circular wards which the country possesses.

### Remarkable Plans and Equipment.

Unfortunately, whoever was responsible for the heating arrangements placed in those wards passed coils which are altogether unsuitable, both in construction and other respects, for use in connection with the circulatory system for warming wards of the type of those to be found in Halifax. There can be no doubt, in our judgment, that although there are central fireplaces, which give a bright appearance to the wards and send forth a considerable amount of warmth, the actual temperature of the wards in the depth of winter, having regard to the window space and the wall space and their superficial contents, must become too low and entail risks to a certain class of patients which ought to be avoided under competent administration in connection with the treatment of patients by a public authority. In a word, the Union Poor-Law Hospital at Halifax may be classed as a modern hospital building, well planned and carefully thought out. Indeed, it is not too much to say as a remarkable fact that the evidence goes to prove that no expense was spared by the Guardians in making the buildings complete and up to date.

### Kitchen, Circular Wards, and Copper Baths.

Thus, as an instance of this, we may refer to the kitchen unit, which is complete and excellent in its appearance, and to the fittings (Slater's), which are of the very best. We have no doubt that the initial cost of this building, though considerable, has been more than repaid already, for the fittings were in excellent order and have stood years of wear without showing any diminution in efficiency or any breakdown. We have already pointed to the excellence of the circular wards. We may mention the baths, which are of copper and have to be kept polished, entailing much labour which could not be provided in an ordinary hospital as it is at Halifax. The polishing is done by some of the inmates, and the whole condition of these baths and, indeed, of the whole establishment reflects great credit upon the matron and her staff of workers. The mortuary, dead-house, and view chamber are unusually good for an establishment like that in question. The Nursing Home provides a separate bedroom for every member of the nursing staff, excellent recreation and class rooms, and vacant space sufficient to enable the nursing staff to be materially increased as circumstances may justify.

### The Flock Pillows.

The bedding and bedsteads throughout the wards are good of their kind, but the flock pillows ought to be thrown out and horsehair bolsters with feather pillows substituted. The reports of the medical officers of the Local Government Board in regard to flock have evidently not yet reached the Guardians and the staff of the Halifax Union Poor-Law Hospital, or flock pillows would have disappeared from this establishment two or three years ago. Taken as a whole it will, therefore, be seen that the equipment of these buildings as they stand is sufficient, though in some respects, *i.e.* the ward furniture, lockers and couches should be changed gradually for better and more suitable types. It is also highly desirable that the heating plant, especially in the circular wards, should be scrapped, and an efficient system of heating introduced into those wards, especially the circular wards. As to the corridors, shut-off doors have been supplied so as to keep the corridors distinct, and in a sense isolated from the wards units, so that it is not material to go to much expense in heating those corridors, which probably require it only in the very coldest winter days and nights.

### Features of the Management.

It was a pleasure to go round with Miss S. E. A. Kidson, the matron, who takes the keenest interest in her work, has considerable practical knowledge, and is undoubtedly not only a good manager but a good administrator too. Her assistant, Miss E. Jones, in her department, exhibited the same good qualities, and the Guardians and the Local Government Board are to be congratulated upon the terms of the Order embodying the regulations of the Halifax Union Poor-Law Hospital, which give to the matron the government and the control of the nurses, the female officers, and servants, with the sick in the wards under the supervision of the medical officer of the hospital. Miss Kidson is, in fact, the mistress of the establishment, which she has brought up to a high state of efficiency. It is governed under rules and conditions, introduced largely by herself, that have contributed materially to the comfort of the inmates and the staff as well as to the general efficiency. This Order grants powers to the Guardians, Article 35, whereby they can, in consultation with the Local Government Board, increase not only the number of the nurses but the scale of salaries to be assigned to each nurse. This is a material provision, because it will simplify the conversion of the Halifax Union Poor-Law Hospital into a State hospital of the first rank directly it is determined, as we hope it shortly will be determined, to convert it into a State hospital for the reasons which we gave fully in The Hospital.

### The Spirit of the Place.

Another inspiring feature of this hospital is the kindly spirit which prevails throughout the whole place, and which characterises the relations between the staff, and the attitude and methods pursued by the hospital committee as representing the Board of Guardians. Nothing could be more considerate, or businesslike, or courteous than the action taken by the present chairman of the hospital committee, Mr. J. W. Miller, to whom we desire to express our personal acknowledgments and best thanks. The same remark applies to Dr. J. F. Woodyatt, the medical officer, who undertakes the treatment of the surgical cases and the operations which become essential in the patients' interests in the course of each year. Our visit was one of the most pleasant, encouraging, and helpful which we have experienced during the inspections we have been engaged upon since 1909.

### Suggestions for Improvement in the Staff Arrangements and its Remuneration.

We think it would be wise of the Guardians to instruct the architect, in conjunction with Dr. Woodyatt, to prepare plans for the improvement of the operation theatre, and to make certain additions to it in the way of an anæsthetic room, a clinical room, improvement in the sterilising arrangements, and generally so as to give the hospital an up-to-date operation unit. We would further suggest that the Guardians would be wise to reorganise the resident medical staff. A hospital of 400 beds should be placed in charge of a medical superintendent of experience, who should have associated with him a resident medical officer as assistant. At present there is only an assistant resident medical officer, and the payment made to that official, £140 a year, is at least £110 too small, having regard to the current rate of pay at present obtainable by qualified members of the medical profession, who would be competent to undertake the duties of this office. We should further be glad to see the title of the medical officer and visiting medical officer changed by giving them the title of visiting surgeon and visiting physician, whilst they retain at least the emoluments which they are at present respectively paid. All these changes would be in the right direction, for they would prepare the way for the promotion of the Halifax Union Poor-Law Hospital into the State hospital of the future, where the majority of the patients will be acute cases, who will have the advantage of the skilled treatment and facilities for recovery which patients enjoy who obtain admission as in-patients to an up-to-date voluntary hospital of the first rank.

### Why Adequate Salaries are Essential.

We are also of the opinion that the salaries paid to the matron and assistant matron are far below the rates which are at present paid to officers in hospitals where the duties are of the important and responsible nature of those discharged especially by Miss Kidson, and, in a less degree, by Miss Jones, her assistant. We think, too, that the salaries of the sisters, after three to five years' service, should be increased to £40 a year. Should the Guardians see their way to adopt these recommendations, Halifax may have the satisfaction of feeling that both the voluntary and the Poor-Law hospitals situated within the borough are of the first rank, and that they fulfil their responsible duties to the community with an efficiency and in a spirit which make them typical of all that is best in hospital life. Finally, we were pleased to see evidence that the residents in Halifax and the neighbourhood do not entirely forget that they may render useful personal service to the inmates of the Union Poor-Law Hospital, and especially to the children, by taking an interest in their cases, by helping them in their troubles, by sending flowers and fruit and other small efforts of that description for the wards and patients, and by visiting the children and taking care that they have an adequate supply of choice picture books and games to enable their days of suffering to be as cheerful and as little irksome as possible. The impression we gained by our inspection of the Halifax Union Poor-Law Hospital was that, if we resided in Halifax or the neighbourhood, we should be proud of this institution and want to lend a hand to do what little we could to maintain and increase the efficiency and usefulness of some of the most interesting and attractive Poor-Law hospital buildings to be found in the country.

The Empty but Well-designed Isolation Block.

The Halifax Union Poor-Law Hospital has in the grounds a well-designed and complete isolation block, which was built for the reception of maternity cases. These buildings must have cost a good round sum. They were empty and had a most neglected look, and had, we believe, never been used, though they were built some thirteen years ago. It would be interesting if the Guardians would instruct their accountant to calculate what actual sum is represented by the expenditure upon these isolation buildings, and to add to it 4 per cent. compound interest for thirteen years. Judged by the plan, the buildings should be capable of fulfilling a most useful and valuable purpose for the sick poor of Halifax who might be suffering from tuberculosis in the first stage. We would venture to suggest that they should be thoroughly cleaned, decorated, and furnished as a small hospital, and that the Guardians should arrange with the Municipal, Health, or Insurance Committees, one or all, to place these buildings at the disposal of the authorities for the purpose of giving immediate treatment to patients in the first stage of tuberculosis. If this step be taken, something will be done to remove these buildings as they exist to-day from affording startling evidence of the relative extravagance in buildings which carried away the Board of Guardians, who are responsible for the acceptance of the original plans from which the Halifax Union Poor-Law Hospital has been built. Seriously, it is a sad pity that this isolation block should be allowed to remain unutilised and unoccupied for one day longer than a little intelligent effort on the part of the Guardians, in combination with the authorities we have named, can effectually prevent.

