# Halifax Union Poor-Law Hospital

**Published:** 1914-12-19

**Authors:** 


### ERRATA.

### HALIFAX UNION POOR-LAW HOSPITAL.

In the article on this hospital in last week's issue, page 243, first column, line 11, the reference should be to The Hospital of February 12, 1910, page 573, instead of to that of October 31 last. On page 244, first column, second paragraph, last line, the reference should be to The Hospital of October 31, 1914, page 113.

